# Use of a Bio-ASP Solution Composed of an Inorganic Alkali, Surfactin, and Xanthan Gum for Enhanced Oil Recovery

**DOI:** 10.1021/acsomega.5c08080

**Published:** 2026-01-03

**Authors:** Landson Soares Marques, Pamela Dias Rodrigues, Verena Filgueiras Borges dos Santos, George Simonelli, Denilson de Jesus Assis, Cristina M. Quintella, Ana Katerine de Carvalho Lima Lobato, Olívia Maria Cordeiro de Oliveira, Luiz Carlos Lobato dos Santos

**Affiliations:** † Oil, Gas, and Biofuels Research Laboratory (PGBio), Postgraduate Program in Chemical Engineering (PPEQ), 28111 Federal University of Bahia (UFBA), R. Prof. Aristides Novis, 2, 2° andar, Federação, CEP, Salvador 40210-630, BA , Brazil; ‡ Institute and Center for Energy and Environment (CIENAM), 28111 Federal University of Bahia (UFBA), R. Av. Adhemar de Barros, s/n, 2° andar, Ondina, CEP, Salvador 40301-110, BA, Brazil; § Food Analysis, Production and Characterization of Biopolymers and Flexible Films Research Group, Postgraduate Program of Chemical Engineering, Federal University of Bahia (UFBA), R. Prof. Aristides Novis, 2, 2° andar, Federação, CEP, Salvador 40210-630, BA, Brazil; ∥ Bioprocess and Biomaterials Engineering Research Group, Postgraduate Program of Chemical Engineering, 67898 Salvador University (UNIFACS), R. Prof. Aristides Novis, 2, 2° andar, Federação, CEP, Salvador 40210-630, BA, Brazil; ⊥ Postgraduate Program in Geochemistry: Petroleum and Environment, Federal University of Bahia (UFBA), R. Av. Adhemar de Barros, s/n, 2° andar, Ondina, CEP, Salvador 40301-110, BA, Brazil

## Abstract

The growing demand for environmentally sustainable enhanced oil recovery technologies has stimulated the development of alternative injection fluids. This study proposes and evaluates a novel biobased alkaline–surfactant–polymer (Bio-ASP) formulation composed of sodium carbonate, the biosurfactant surfactin, and the biopolymer xanthan gum, all derived from renewable resources. A full factorial 2^3^ experimental design with three central replicates (11 core flooding experiments) was employed to investigate the individual and synergistic effects of these components on sandstone oil reservoirs. The Bio-ASP solutions were characterized in terms of the density, pH, interfacial tension, and viscosity. Results revealed that the surfactin concentration was the main factor in reducing interfacial tension, while xanthan gum primarily controlled viscosity and mobility, both significantly improving the oil recovery factor. The optimized Bio-ASP system achieved up to 63% tertiary oil recovery, outperforming conventional ASP systems reported in the literature. These findings highlight the novelty and technical viability of a fully biobased ASP system, offering a more sustainable and scalable alternative for efficient exploitation of mature or low-productivity reservoirs.

## Graphical abstract

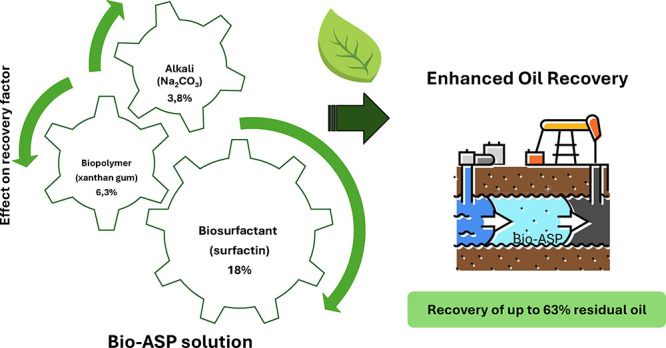

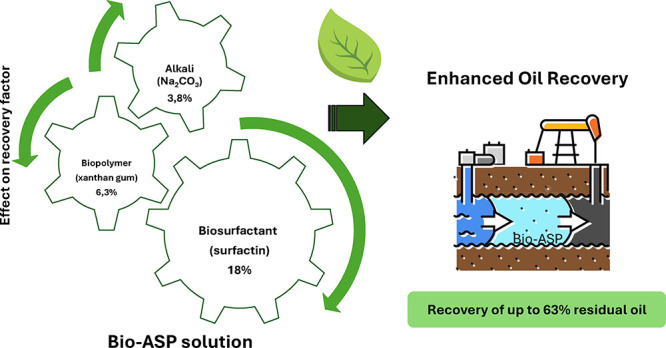

## 1 Introduction

Environmentally friendly enhanced oil recovery (EOR) methods, including microbially enhanced oil recovery (MEOR), low-salinity water flooding, polymer flooding, macromolecular compounds, and their combinations with surfactants and water-alternating-gas (WAG) injection, have shown exponential growth in technological development in recent years.^1^

This trend reflects the growing need to develop oil recovery technologies with a reduced environmental footprint aligned with the goals of a responsible energy transition. Such efforts aim to mitigate the environmental impacts of oil production^2^ while recognizing the global dependence on fossil fuels as a primary energy source for the foreseeable future.^3,4^

Among the various EOR techniques, the injection of alkali/surfactant/polymer (ASP) mixtures stands out as one of the most effective and promising strategies. The synergistic interactions among alkali, surfactant, and polymer components contribute to improved oil recovery by enhancing emulsification, reducing interfacial tension (IFT), and increasing sweep efficiency, thereby boosting overall injection performance.^5^ Despite its potential, the displacement mechanisms involved in ASP flooding, as well as the specific roles of each component in the oil recovery process, remain subjects of continued research. Furthermore, the surfactants and polymers typically used in ASP formulations are often expensive and resistant to environmental degradation, raising economic and ecological concerns regarding their widespread application.

Recent studies have demonstrated that the combined effects of polymer viscoelasticity and wettability alteration play a decisive role in improving oil recovery during flooding processes. Zhong et al.^6^ reported that these two mechanisms act synergistically to enhance the displacement of oil droplets in porous media, leading to higher recovery efficiencies. Similarly, Rock et al.^7^ reviewed the influence of polymer elasticity in porous media and highlighted its ability to reduce viscous fingering and improve sweep efficiency. Furthermore, Golab et al.^8^ provided additional evidence on the effectiveness of polymeric surfactants in reducing IFT and altering rock wettability, achieving recovery rates of up to 84% of the original oil in place. Collectively, these findings reinforce the importance of integrating viscoelastic and interfacial mechanisms to advance the performance of chemical flooding strategies in EOR.

In light of these limitations, microbial enhanced oil recovery (MEOR) has emerged as one of the most promising alternatives, offering a simpler process, lower operational costs, and significantly reduced environmental impact compared to conventional chemical EOR methods.^9^ Microbial activity can be controlled either ex situ or in situ, and it can be applied to a variety of reservoirs, including those with oils that exhibit diverse physical and chemical properties. Additionally, MEOR is effective in low-permeability reservoirs, where it helps to reduce water flow within the reservoirs, thereby minimizing excess water production.^10,11^

A variety of bioproducts can be derived from bacteria, including biosurfactants, biopolymers, bioacids, biogases, and bioalcohols. Biosurfactants, in particular, enhance the mobility of trapped oil due to their surface-active properties, which reduce the IFT between crude oil and water and, in many cases, can modify the wettability of reservoir rocks. Biopolymers, on the other hand, improve mobility control and sweep efficiency by increasing the viscosity of the injected water, thereby reducing its flow rate and enhancing displacement effectiveness.^12–11^

Biosurfactants offer advantages, such as high interfacial activity, biodegradability, and potential for wettability modification. However, they still present relatively high production costs, sensitivity to extreme reservoir conditions, and challenges related to the long-term stability. Meanwhile, biopolymers such as xanthan gum significantly contribute to viscosity enhancement and mobility control, being biodegradable and derived from renewable sources. Nevertheless, they may experience retention and adsorption in porous media, which can reduce their effective performance in EOR applications. Overall, while biosurfactants and biopolymers exhibit distinct mechanisms of action in EOR processes, their combined use can lead to synergistic effects, balancing the limitations of each component and enhancing both displacement efficiency and environmental performance.^11,12^

The application of biosurfactants in microbial enhanced oil recovery (MEOR) encompasses several strategies, including the injection of biosurfactant-producing microorganisms into the reservoir followed by in situ propagation; the introduction of nutrients to stimulate the growth of indigenous biosurfactant-producing microorganisms; and the production of biosurfactants in biorreactors, which are then injected into the reservoir.^14^

In addition to biosurfactants, biopolymers possess numerous properties that make them suitable for applications across various industrial sectors, including food, cosmetics, and advanced oil recovery, among others.

Biopolymers are polymeric materials that are structurally classified as polysaccharides, polyesters, or polyamides.^15^ The primary raw material for biopolymer production is a renewable carbon source, typically a carbohydrate derived from large-scale commercial crops such as sugarcane, corn, potatoes, wheat, and beets, or vegetable oils extracted from soybeans, sunflowers, palm, or other oil-bearing plants.^16^

A major advantage of biopolymers is their biodegradability in the environment, which occurs because of the activity of naturally occurring microorganisms such as bacteria, fungi, and algae.^17^ Therefore, biopolymers are gaining increasing attention due to their unique properties, which contribute to an extended material lifespan while simultaneously facilitating environmentally friendly disposal, making them a promising alternative to conventional polymers.

Although biosurfactants and biopolymers offer clear environmental and economic advantages, some limitations must also be considered for their large-scale application. Biosurfactants such as surfactin often present relatively high production costs compared to synthetic surfactants, mainly due to the complexity of fermentation and downstream recovery processes. They may also suffer from stability issues under extreme reservoir conditions such as salinity, pH, or temperature. Similarly, biopolymers like xanthan gum, while effective in improving viscosity, can experience shear degradation, susceptibility to microbial attack, and potential adsorption or retention in porous media, which may reduce injectivity and long-term efficiency. These factors highlight the importance of further optimization and scale-up strategies to ensure that biobased ASP formulations are both technically viable and economically competitive.

Despite the environmental and technical advantages, the economic viability of Bio-ASP still presents a challenge. High concentrations of surfactin (up to 10,000 mg·L^–1^) and xanthan gum (up to 5000 mg·L^–1^) can generate significant costs at field scale. Production strategies using residual substrates, optimization of the fermentation process, and biomass recovery may reduce costs and make practical applications more competitive.

ASP flooding improves oil recovery through a combination of physicochemical mechanisms. The alkali component reacts with acidic crude oil components to generate in situ surfactants, which contribute to additional IFT reduction and help stabilize the emulsions. Surfactants reduce IFT directly, overcoming capillary forces that trap residual oil and promoting the formation of microemulsions at the oil–water interface. Polymers primarily increase the viscosity of the displacing fluid, improving the mobility ratio and sweep efficiency while reducing the level of viscous fingering and channeling. In addition, synergistic interactions among these components can lead to enhanced stability of the injected solution, improved wettability alteration, and the mitigation of surfactant adsorption onto reservoir rock surfaces. Together, these mechanisms make ASP flooding one of the most effective chemical EOR techniques reported in the literature.^5,9^

Despite the recognized potential of alkaline–surfactant–polymer (ASP) flooding, most reported formulations still rely on synthetic surfactants and polymers that are costly, environmentally persistent, and sometimes inefficient under reservoir conditions. Studies integrating biosurfactants and biopolymers into ASP systems remain scarce, and the synergistic contributions of these components to oil displacement have not been systematically quantified. In this context, the present study addresses this research gap by developing and optimizing a biobased ASP formulation (Bio-ASP) composed of sodium carbonate (alkali), surfactin (biosurfactant), and xanthan gum (biopolymer). Using a full factorial experimental design, the individual and interactive effects of these components were evaluated to establish the most influential variables and maximize oil recovery efficiency.

## 2 Materials and Methods

### 2.1 Oil

The crude oil employed in the displacement experiments, supplied by Petrobras, is sourced from the Carmópolis field located in Sergipe, Brazil. Its physicochemical properties are detailed in Table 1.

**Table 1:** Analyzed Physicochemical Properties of Crude Oil from the Carmópolis Field, Sergipe, Brazil

physicochemical properties	defined value	technical standard
pour point (°C)	20	ASTM D97
specific gravity at 20 °C (g.mL^–1^)	0.900 ± 0.005	ASTM D-5002
specific gravity at 60 °C (g.mL^–1^)	0.890 ± 0.005	ASTM D-5002
viscosity at 60 °C (cP)	42.43 ± 0.03	ASTM D-445
BSW (%)	50	NBR-14647
API grade	25.7	ASTM D-5002

### 2.2 Production of the Biosurfactant Surfactin

The biosurfactant was produced using the microorganism *Bacillus subtilis* (UFPEDA86), with anhydrous d-glucose P.A. (Synth brand) employed as the carbon source during the fermentation process. The production protocol was structured into the following stages: strain activation, preinoculum and inoculum preparation, biosurfactant production, and extraction of the crude surfactin. The methodology for surfactin production was adapted from the procedure described by Soares et al.^18^

The media used for both the preinoculum and inoculum had the same composition: 20.0 g·L^–1^ of anhydrous d-glucose P.A., 3.0 g·L^–1^ of KH_2_PO_4_, 7.0 g·L^–1^ of K_2_HPO_4_, 0.2 g·L^–1^ of MgSO_4_·7H_2_O, 1.0 g·L^–1^ of (NH_4_)_2_SO_4_, and 1.0 g·L^–1^ of yeast extract. After combining the components, the pH of the medium was adjusted to 6.8 using either 1 M NaOH or 1 M HCl, followed by sterilization by autoclaving at 121 °C and 1 atm for 15 min under moist heat.

The preinoculum was prepared by transferring three loops of culture from a slant tube into a 125 mL Erlenmeyer flask containing 30 mL of sterile medium. The flask was then incubated at 37 °C with orbital shaking at 200 rpm for 6 h. For inoculum preparation, a 250 mL Erlenmeyer flask containing 50 mL of the culture medium was used. An aliquot corresponding to 10% (v/v) of the inoculum (5 mL) was transferred from the preinoculum into the flask, which was subsequently incubated under the same conditions (37 °C, 200 rpm) for approximately 16 h.

Under aseptic conditions in a laminar flow hood, sterilized Erlenmeyer flasks containing the fermentation medium were inoculated by transferring an aliquot equivalent to 10% (v/v) of the culture volume, i.e., 5 mL per flask. Following inoculation, biosurfactant production was carried out via submerged fermentation in an orbital shaker incubator at 37 °C and 200 rpm for 96 h.

Initially, the supernatants were acidified with 3 M HCl to a final pH of 2.0. The acidified solutions were then stored at 4 °C for 24 h to promote biosurfactant precipitation. The resulting precipitates (pellet) were separated from the supernatant by centrifugation at 4000 rpm and 20 °C for 10 min, using preweighed and labeled test tubes. The precipitates were subsequently dried in an oven at 55 °C for 24 h to remove residual acid.

The extracted biosurfactant was not subjected to purification; the resulting material—crude surfactin—was used directly in the preparation of the Bio-ASP formulation. Although the biosurfactant was not subjected to purification, we standardized the culture medium and maintained strict control of the bioprocess, ensuring preservation of the physicochemical characteristics of the crude biosurfactant across different batches. Surface tension measurements and determination of the critical micelle concentration (CMC) confirmed the reproducibility required for application in bio-ASP. Furthermore, as reported by Charoentanaworakun et al.^19^ and Câmara et al.,^20^ the CMC is a widely accepted parameter to assess biosurfactant efficiency, as it reflects the minimum concentration required to produce a significant reduction in the surface tension of water.

The choice not to purify the surfactin is technically justified since purification steps substantially increase the cost of the biosurfactant, reducing its feasibility for field-scale applications. By using surfactin in its crude form, combined with rigorous bioprocess control and standardized cultivation conditions, production costs are reduced, thereby enhancing the industrial applicability of the technology proposed in this work.

### 2.3 Production of the Biopolymer Xanthan Gum

Xanthan gum was supplied by the Laboratory of Fish and Applied Chromatography (LAPESCA—UFBA). The biopolymer was produced by *Xanthomonas campestris* mangiferaeindicae 2103 through the consumption of crude glycerol, a coproduct of biodiesel production, which was used as a substrate for microbial growth and biopolymer synthesis.

The xanthan gum used in this study was produced under conditions similar to those reported in previous work^16^ and exhibited an average molecular weight of approximately 2.0 × 10^6^ Da, which falls within the typical range (2–5 × 10^6^ Da) reported for xanthan produced by *Xanthomonas campestris* strains.^12,16^ The methodology for xanthan gum production is described in Assis et al.^21^

### 2.4 Alkaline Selection

Sodium carbonate (Na_2_CO_3_) was selected as the alkali component of the Bio-ASP formulation because of its proven efficiency in reducing IFT and its wide application in conventional ASP flooding. Compared to stronger alkalis such as sodium hydroxide, Na_2_CO_3_ is less corrosive and easier to handle in field operations while still providing sufficient alkalinity to react with acidic crude oil components and generate in situ surfactants. In addition, it contributes to stabilizing the pH of the injected fluid within a favorable range for biosurfactant activity, thereby reducing the risk of surfactant precipitation or degradation. Its relatively low cost and availability also make it a practical choice for large-scale implementation of Bio-ASP systems.^22^

### 2.5 Recovery Factor Measures

The experimental procedure employed for the displacement tests and recovery factor measurements is an adaptation of the method described by Ramos et al.,^23^ Marques et al.^24^ and Quintella et al.^25^

A relevant Core Holder system was employed and mounted inside an oven that maintained a fixed temperature of 60 ± 2 °C. In this system, the sandstone core sample was confined under a pressure of 1000 psi. Fluids were injected using an HPLC pump (Jasco, model PU-4087) at a constant flow rate of 1.0 mL·min^–1^.

The sandstone plugs were sourced from the Botucatu Formation, Brazil, with a nominal permeability ranging from 300 to 400 mD. Their dimensions were adapted to a diameter of 3.8 cm and a length of 7.0 cm.

The degassing of the rock plugs was carried out by immersing each core in synthetic formation water (40,000 mg·L^–1^ of NaCl and 13,000 mg·L^–1^ of Na_2_SO_4_) under a vacuum of 1 × 10^–3^ Torr (using an Edwards RV5 vacuum pump) for a minimum of 5 h, with no gas release observed at the end. The pore volume of each plug was determined by calculating the mass difference between the dry plug and the saturated plug divided by the specific gravity of the synthetic formation water.

Sequence of fluid injection:

1. Injection of 1 pore volume (PV) of synthetic formation water to ensure complete saturation of the core sample.
2. Injection of 2 PV of crude oil to displace the formation water and establish residual water saturation.
3. Injection of synthetic saline water until residual oil saturation (Sor) was achieved.
4. Injection of Bio-ASP solution to further displace the remaining oil, resulting in a new residual oil saturation.

Throughout each test, the produced fluids were collected at every half-pore volume injected and placed in preweighed containers, allowing for a mass balance to be performed. After the completion of each injection test, the produced water, i.e., the aqueous phase, was separated from the oil phase, and both phases were weighed.

In practical field applications, the water quality used for polymer makeup is a critical operational factor, particularly for xanthan gum systems. In this study, the core saturation steps were performed using synthetic formation brine (40,000 mg·L^–1^ NaCl and 13,000 mg·L^–1^ Na_2_SO_4_), while the Bio-ASP formulation was prepared in distilled water to prevent polymer precipitation and surfactant destabilization.

Furthermore, biopolymers, such as xanthan gum, may be susceptible to bacterial degradation under reservoir conditions. Field-scale implementations typically require the addition of biocides or thermal stabilization strategies to ensure polymer integrity along the injection line and during the reservoir residence time. This operational consideration should be incorporated into future pilot-scale evaluations.

### 2.6 Design of Experiments (DOE)—2^3^ Factorial Design with Triplicate Central Point

In order to analyze the effect of the concentration of each component of the Bio-ASP solution on the oil recovery factor, a full factorial experimental design of the 2^3^ type was conducted with three replicates at the central point. Table 2 presents the factor levels and the concentration of each component used in the planned displacement tests.

**Table 2:** DOE Matrix for the Injection Tests

		concentration of components (mg·L^–1^)		
test	solution	Na_2_CO_3_	crude surfactin	xanthan gum
1	Bio-ASP1	1200 (−1)	3000 (−1)	1000 (−1)
2	Bio-ASP2	1200 (−1)	10,000 (+1)	1000 (−1)
3	Bio-ASP3	1200 (−1)	3000 (−1)	5000 (+1)
4	Bio-ASP4	1200 (−1)	10,000 (+1)	5000 (+1)
5	Bio-ASP5	4500 (+1)	3000 (−1)	1000 (−1)
6	Bio-ASP6	4500 (+1)	10,000 (+1)	1000 (−1)
7	Bio-ASP7	4500 (+1)	3000 (−1)	5000 (+1)
8	Bio-ASP8	4500 (+1)	10,000 (+1)	5000 (+1)
9	Bio-ASP9	2850 (0)	6500 (0)	3000 (0)
10	Bio-ASP10	2850 (0)	6500 (0)	3000 (0)
11	Bio-ASP11	2850 (0)	6500 (0)	3000 (0)

The experimental values obtained for the EOR recovery factor using the Bio-ASP solutions were statistically analyzed using the Statistica software, version 7.0, through multiple regression analysis. This statistical evaluation allowed for the development of a mathematical model for the recovery factor. The multiple regression was performed using coded independent variables, which were applied to standardize the actual factor values and prevent potential bias in the statistical analysis due to the diverse ranges of the factors. An Analysis of Variance (ANOVA) was conducted to assess whether the mathematical model obtained from multiple regressions was statistically significant. A confidence level of 95% was adopted for all of the statistical analyses.

The regression was performed by using the results from the injection tests conducted in the laboratory. Through this analysis, it was possible to obtain a mathematical model, expressed as an equation, that demonstrates how the independent variables (concentrations) influence the recovery factor. The regression-derived equation was used to plot the response surfaces. eq 1 presents a first-order mathematical model with interactions for a design involving three independent variables. In this study, only statistically significant independent variables were included in the final model following regression analysis (eq 1).

$$\gamma =\alpha_{0}+\alpha_{1}X_{1}+\alpha_{2}X_{2}+\alpha_{3}X_{3}+\alpha_{12}X_{1}X_{2}+\alpha_{13}X_{1}X_{3}+\alpha_{23}X_{2}X_{3}$$ 1

where *X*_1_ = alkali, *X*_2_ = biosurfactant, and *X*_3_ = biopolymer are the independent variables of the model; α_0_ is the intercept coefficient; α_1_, α_2_, and α_3_ are the coefficients corresponding to the linear effects; and α_12_, α_13_, and α_2__3_ are the coefficients corresponding to the interaction effects among the variables.

### 2.7 Preparation of the Bio-ASP Solution

The Bio-ASP solutions were prepared using sodium carbonate (Na_2_CO_3_) analytical grade Lumatom as the alkali, produced surfactin as the biosurfactant, and produced xanthan gum as the biopolymer.

To ensure complete homogenization of the solutions, the appropriate amounts of each component (alkali, biosurfactant, and biopolymer) were added according to the proportions defined in the experimental design (Table 2) to 100 mL of distilled water. The mixture was then stirred at 1500 rpm using an electronic mechanical stirrer (Quimis, Q235) for 5 h. Subsequently, the solution was centrifuged at 2200 rpm for 15 min to remove air bubbles incorporated during agitation.

### 2.8 Physicochemical Characterization of the Bio-ASP Solutions

The Bio-ASP solutions used in the tests were characterized by viscosity, IFT, specific mass, and pH.

The rheological behavior of the Bio-ASP solution was determined using the Physica rheometer, model MCR 501, from Anton Paar, with concentric cone geometry, in accordance with the ASTM D-445 standard. Since these are non-Newtonian fluids, the experimental apparent viscosity data collected from the rheometer were mathematically regressed with the assistance of the Rheoplus software, provided by Anton Paar, employing the Ostwald de Waele model. The apparent viscosities, presented in Table 4, were calculated at a shear rate of 100 s^–1^.

The IFT measurements between crude oil and the Bio-ASP solutions were performed using the pendant drop method on a DataPhysics tensiometer, model OCA 15 plus. The equipment is equipped with a temperature controller, and the measurements were carried out at 60 ± 2 °C. Each Bio-ASP solution was placed in a quartz cuvette, and the oil drop was formed within the solution through an inverted needle. A high-resolution camera was used to capture the images, and the embedded software was employed to analyze the drop dimensions and calculate the IFT by using the Yang-Laplace equation. Five well-formed oil drops were analyzed in each of the solutions, and the average IFT value was used as the response value.

The specific gravity of the Bio-ASP solution was measured by using a digital densimeter (Anton Paar, DMA-5000) in accordance with ASTM D5002. The pH was determined with a pH meter (TECNOPON, model mPA-210).

## 3 Results and Discussion

### 3.1 Physical-Chemical Characterization of Bio-ASP Solutions

Table 3 summarizes the results of the physical-chemical characterizations performed.

**Table 3:** Physicochemical Properties of the Bio-ASP Solutions Used in the Injection Tests

solution	Na_2_CO_3_ (mg·L^–1^)	surfactin (mg·L^–1^)	xanthan gum (mg·L^–1^)	density (g·mL^–1^)	pH	interfacial tension (mN·m^–1^)	apparent viscosity (cP)
Bio-ASP1	1200	3000	1000	1.089 ± 0.003	8.70 ± 0.03	1.23 ± 0.02	0.810
Bio-ASP2	1200	10,000	1000	1.183 ± 0.003	8.70 ± 0.03	0.85 ± 0.05	1.630
Bio-ASP3	1200	3000	5000	1.132 ± 0.003	8.70 ± 0.03	1.14 ± 0.02	3.120
Bio-ASP4	1200	10,000	5000	1.201 ± 0.003	8.70 ± 0.03	0.88 ± 0.01	12.900
Bio-ASP5	4500	3000	1000	1.089 ± 0.003	8.90 ± 0.03	1.13 ± 0.02	0.940
Bio-ASP6	4500	10,000	1000	1.183 ± 0.003	8.90 ± 0.03	0.81 ± 0.03	1.960
Bio-ASP7	4500	3000	5000	1.132 ± 0.003	8.90 ± 0.03	1.20 ± 0.09	3.220
Bio-ASP8	4500	10,000	5000	1.201 ± 0.003	8.90 ± 0.03	0.9 ± 0.1	13.100
Bio-ASP9	2850	6500	3000	1.142 ± 0.003	8.70 ± 0.03	1.1 ± 0.1	5.730
Bio-ASP10	2850	6500	3000	1.142 ± 0.003	8.70 ± 0.03	1.1 ± 0.1	5.730
Bio-ASP11	2850	6500	3000	1.142 ± 0.003	8.70 ± 0.03	1.1 ± 0.1	5.730

The physicochemical characterization of the Bio-ASP solutions revealed how the concentrations of alkali (Na_2_CO_3_), biosurfactant (surfactin), and biopolymer (xanthan gum) influence key fluid properties—namely, density, pH, IFT, and apparent viscosity—each of which plays a critical role in EOR efficiency.

The density of the Bio-ASP solutions ranged from 1.089 to 1.201 g·mL^–1^. Higher densities were observed in solutions with elevated concentrations of biosurfactant and biopolymer (e.g., Bio-ASP4 and Bio-ASP8), indicating the additive mass contributions of these components to the fluid. This is particularly relevant in mobility control and flow assurance during injection.

IFT values were strongly influenced by the biosurfactant concentration. Solutions with lower surfactin content (3000 mg·L^–1^) presented higher IFTs (up to 1.23 mN·m^–1^), while those with 10,000 mg·L^–1^ of surfactin achieved significantly reduced IFTs (as low as 0.81 mN·m^–1^). This reduction is essential for mobilizing trapped oil, as it diminishes capillary forces and facilitates emulsification. Notably, the lowest IFT was recorded in Bio-ASP6 (0.81 mN·m^–1^), which combines high alkali and high biosurfactant concentrations.

Apparent viscosity values varied widely, from 0.810 to 13.100 cP, and were clearly governed by the concentration of xanthan gum. Formulations containing 5000 mg·L^–1^ of biopolymer (e.g., Bio-ASP4 and Bio-ASP8) showed the highest viscosities, reinforcing xanthan gum’s effectiveness in increasing fluid resistance to flow. This contributes directly to the mobility control and sweep efficiency during the displacement process. In contrast, solutions with low biopolymer concentrations (1000 mg·L^–1^) exhibited significantly lower viscosities.

However, a comparative assessment indicates that, at a fixed xanthan concentration (1000 mg·L^–1^), increasing the surfactin level from 3000 mg·L^–1^ (Bio-ASP1:0.810 cP) to 10,000 mg·L^–1^ (Bio-ASP2:1.630 cP) yields a pronounced 101% increase in apparent viscosity. This response is indicative of enhanced surfactin–polymer associative interactions and potential microstructural organization within the solution, which collectively contribute to more effective mobility control.

The most favorable combination of properties was found in Bio-ASP8, which exhibited high density (1.201 g·mL^–1^), high pH (8,90), low IFT (0.9 mN·m^–1^), and high apparent viscosity (13.100 cP). These characteristics are indicative of an optimized EOR fluid, balancing interfacial activity and flow control. Conversely, Bio-ASP1, with all components at their lowest concentrations, displayed the least favorable profile with high IFT and low viscosity.

These results underscore the importance of adjusting component concentrations in a synergistic manner to tailor fluid properties to reservoir needs. In particular, the dominance of biosurfactant in reducing IFT and biopolymer in enhancing viscosity aligns well with the statistical analysis discussed later in the article.

### 3.2 Measurement of Recovery Factor

Table 4 presents the recovery factors in the conventional water injection stage (RF_W_) and in the nonconventional Bio-ASP injection stage (RF_A_) of the Core Holder displacement tests. The recovery factors during water injection ranged from 30 to 36% of the Original Oil in Place (OOIP). These values fall within the range previously reported by Kumar et al.^26^

**Table 4:** Oil Recovery Factors during Water and Bio-ASP Injection Stages in Core Displacement Tests with Rock Samples^a^

fluid	RF_W_ (%)	RF_A_ (%)	RF (%)	Δ*P* (psi)	*S*_OR_ (%)	*N*_C_
Bio-ASP1	34 ± 2	36 ± 1	70 ± 3	250	19.90	4.87 × 10^–6^
Bio-ASP2	32 ± 2	52 ± 1	84 ± 3	240	8.35	1.42 × 10^–5^
Bio-ASP3	31 ± 2	44 ± 1	75 ± 3	320	9.59	2.02 × 10^–5^
Bio-ASP4	31 ± 2	62 ± 1	92 ± 3	430	0.67	1.08 × 10^–4^
Bio-ASP5	32 ± 2	40 ± 1	72 ± 3	250	18.70	6.15 × 10^–6^
Bio-ASP6	31 ± 2	61 ± 1	92 ± 3	240	0.12	1.79 × 10^–5^
Bio-ASP7	33 ± 2	45 ± 1	78 ± 3	330	7.26	1.98 × 10^–5^
Bio-ASP8	32 ± 2	63 ± 1	95 ± 3	440	2.21	1.08 × 10^–4^
Bio-ASP9	33 ± 2	53 ± 1	86 ± 3	290	5.83	3.85 × 10^–5^
Bio-ASP10	32 ± 2	53 ± 1	85 ± 3	280	6.74	3.85 × 10^–5^
Bio-ASP11	35 ± 2	56 ± 1	91 ± 3	290	1.08	3.85 × 10^–^^5^

a (RF_W_ = Water recovery factor; RF_A_ = Bio-ASP recovery factor; RF = total recovery factor; Δ*P* = variation of the injection pressure of the Bio-ASP fluid; *S*_OR_ = residual oil saturation after recovery with the Bio-ASP fluid; *N*_C_ = capillary number).

The analysis of the Δ*P* values indicates that the variation in injection pressure reflects both the mobility and the stability of the chemical slug within the porous medium, being influenced by the rheological and interfacial properties of the Bio-ASP formulations. More viscous solutions exhibited higher Δ*P* and improved sweep capability, while systems with a more stable Δ*P* demonstrated an adequate balance between viscosity and flowability.

The capillary number was calculated as *N*_C_ = μv/σ, where μ is the apparent viscosity of the solution (Pa·s), *v* is the superficial injection velocity (m·s^–1^), and σ is the oil/solution IFT (N·m^–1^). Figure 1 shows the relationship between log (*N*_c_) and Sor, highlighting how the combined effects of solution viscosity and IFT reduction govern the mobilization of residual oil, enabling the identification of the regime in which capillary forces are overcome by the mobility of the displacing phase.

**Figure 1:**
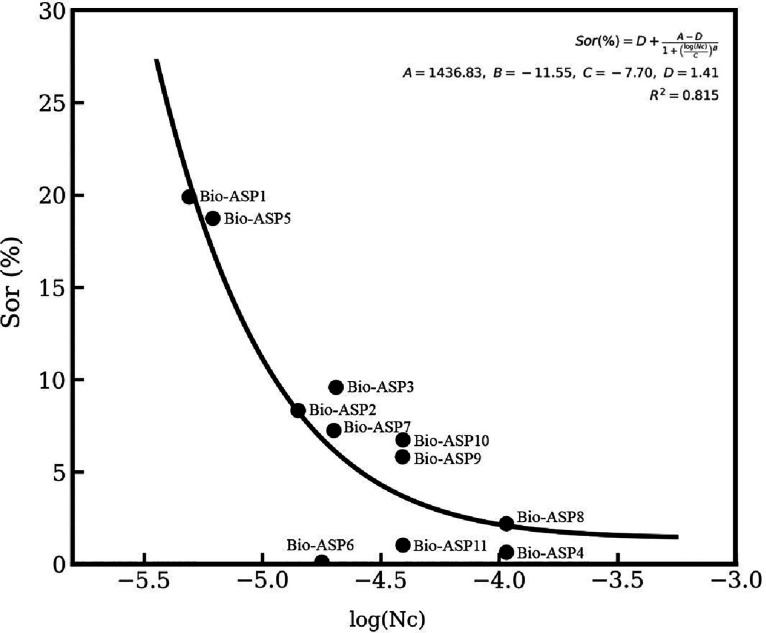
Capillary desaturation curve showing the relationship between log (*N*_c_) and residual oil saturation (Sor) in the core-flooding tests.

The increase in apparent viscosity, as shown in Table 3 and reinforced by the indirect analysis of surfactin–xanthan synergy, was important for raising the capillary number. Although the IFT did not reach the ultralow regime, the increase in the viscous term of the capillary number compensated for this limitation, allowing oil mobilization to be dominated by mobility control and polymer-induced viscoelasticity, as suggested by previous studies.

A comparative interpretation of the Sor × log (*N*_c_) desaturation curve demonstrates that the evaluated formulations consistently follow the behavior described by the classical correlations reported in the literature.^27–29^ In the region of very low capillary numbers (log *N*_c_ < −5), the Bio-ASP1 and Bio-ASP5 solutions, with *N*_c_ on the order of 10^–6^, exhibit limited desaturation, which is fully consistent with the literature stating that virtually no mobilization of residual oil occurs below the critical *N*_c_ (∼10^–5^). As the formulations approach the critical threshold (−5 ≤ log *N*_c_ ≤ −4.5), as observed for Bio-ASP2, Bio-ASP3, Bio-ASP7, Bio-ASP9, Bio-ASP10, and Bio-ASP11, a significant reduction in Sor is evident, faithfully reproducing the transition predicted in the original curves from previous works.^29^ For *N*_c_ above the critical regime (log *N*_c_ > −4.5), the Bio-ASP4 and Bio-ASP8 solutions, with *N*_c_ close to 10^–4^, present Sor values below 3%, a behavior compatible with the viscous-flow regime in which the literature reports residual values near 1%.^28,29^ These results confirm that the Bio-ASP formulations follow the universal trend of the Sor–*N*_c_ relationship, demonstrating their effectiveness in mobilizing residual oil in porous media.

A strong correlation was observed between log (*N*_c_) and residual oil saturation (Sor), with *R* ^2^ ≈ 0.82, indicating that the increase in the capillary number (greater viscous/velocity contribution relative to IFT) favored the reduction of Sor in the evaluated samples. This behavior suggests that, under the experimental conditions adopted, the relative mobility of the aqueous phase and the alteration of capillary forces play a cooperative role in the release of residual oil, supporting the hypothesis of a synergistic action between the rheological effects (xanthan) and the interfacial effects (surfactin).

The role of the surfactin concentration in oil recovery extends beyond the simple reduction of IFT. Although the IFT values obtained did not reach the ultralow regime, the enhanced recovery observed is associated with an integrated mechanism. Increasing the surfactin concentration contributed to altering rock wettability toward a state more favorable to the displacement of the oil phase, promoted the formation of sufficiently stable oil-in-water emulsions capable of mobilizing retained oil, and increased the apparent viscosity of the solution when in synergy with xanthan, resulting in improved mobility control of the chemical slug. Additionally, the partial reduction of capillary forces, even if moderate, indicates that oil desaturation does not rely solely on achieving ultralow IFT but rather on the combined interaction of rheological, interfacial, and wettability-modifying effects. This combination of mechanisms explains the significant influence of surfactin on the recovery performance observed.

### 3.3 Response Surface

Through statistical analyses, it was possible to evaluate the effects of varying the concentration of the compounds in the injection fluids used in the enhanced recovery tests, as shown in Table 5.

**Table 5:** Statistical Analysis of the Individual and Synergistic Effects of Alkali, Biosurfactant, and Biopolymer on the Experimental Response in the Enhanced Oil Recovery Factor

			confidence limit	
factor	effect	*p*-value	–95%	+95%
alkali	3.82500	0.004776	2.68324	4.96676
biosurfactant	18.10000	0.000215	16.95824	19.24176
biopolymer	6.32500	0.001756	5.18324	7.46676
alkali × biosurfactant	1.17500	0.047406	0.03324	2.31676
alkali × biopolymer	–2.55000	0.010656	–3.69176	–1.40824
biosurfactant × biopolymer	–0.14500	0.639587	–1.28676	0.99676

In Table 5, it can be observed that the largest main effects were those of the biosurfactant concentration (18.1%) and the biopolymer concentration (6.325%). Both effects are statistically significant (*p* < 0.05). The biosurfactant concentration plays a predominant role in increasing or decreasing the recovered fraction.

Based on the total recovered fraction, an increase in the alkali concentration from the low to the high level (1200–4500 mg L^–1^) increases the recovered fraction by only 1.82%. In contrast, for the biosurfactant concentration, an increase from the low to the high level (3000–10,000 mg L^–1^) results in an 18.1% increase in the recovered fraction.

Observing the first-order interactions, the interaction between alkali concentration and biosurfactant concentration was statistically significant (*p*-value <0.05), with an effect of 1.175. The interactions between alkali × biopolymer and alkali × biosurfactant also have *p*-values lower than 0.05, making them statistically significant as well.

According to the effects, if both the alkali concentration and the biopolymer concentration remain at low levels (1200–1000 mg L^–1^, respectively) or high levels (4500 and 5000 mg L^–1^, respectively), the recovered fraction will decrease by 2.55%. Similarly, if both, the biosurfactant concentration and the biopolymer concentration remain at low levels (3000 and 1000 mg L^–1^, respectively) or high levels (10,000 and 5000 mg L^–1^, respectively), the recovered fraction will decrease by 0.145%.

The p-value of the biosurfactant concentration is lower than 0.05 and smaller than the p-values of the alkali and biopolymer, indicating that the concentration of this compound is the most significant independent variable across all experiments. The influence of the Bio-ASP solution components on the EOR fraction is presented in hierarchical order through the Pareto chart, as shown in Figure 2A. This chart was constructed from the standardized effects obtained in the factorial design analysis, where the bars represent the magnitude and statistical significance of both the main and interaction effects of alkali, biosurfactant, and biopolymer concentrations. The effects were calculated from the regression coefficients of the coded variables, and statistical significance was assessed at the 95% confidence level.

**Figure 2:**
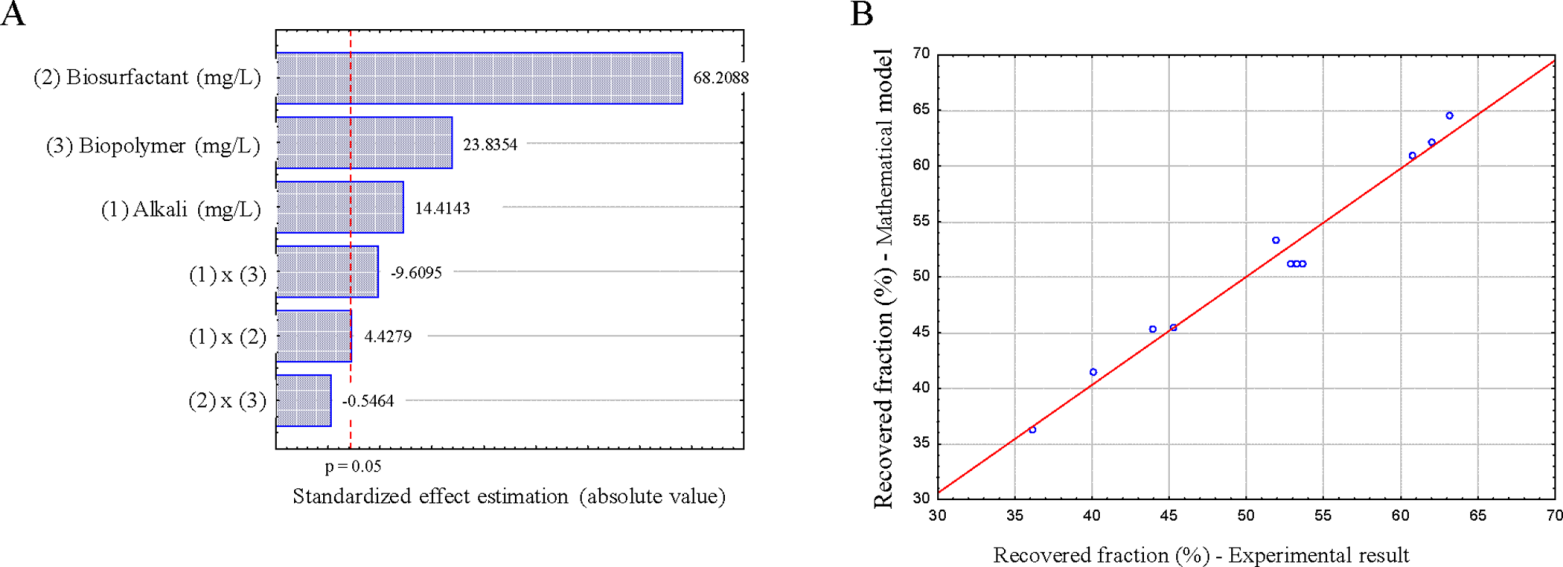
(A) Pareto chart illustrating the magnitude and statistical significance of the main and interactive effects of the Bio-ASP solution components on the oil recovery factor. The chart was constructed from the standardized effects obtained from the factorial design analysis. (B) Correlation plot demonstrating the strong relationship between the experimental results (recovered fraction) and the values predicted by the mathematical model obtained from the regression analysis.

The concentrations of the biosurfactant and biopolymer are the most statistically significant independent variables. In contrast, the alkali concentration and its interactions with the other components of the Bio-ASP fluid did not yield *p*-values as low as those of the others, indicating a lower influence on the response variable.

Equation 2 represents the mathematical model with a *R* ^2^ of approximately 97.31%. The coefficient of determination indicates that 97.31% of the variation in the recovered fraction is explained by the variation in the concentrations of alkali, biosurfactant, and biopolymer.

$$F_{\mathrm{RA}}(\%)=51.208+1.912\;\mathrm{AC}+9.050\;\mathrm{BSC}+3.163\;\mathrm{BPC}+0.588\;\mathrm{AC}\times \mathrm{BSC}-1.275\;\mathrm{AC}\times \mathrm{BPC}$$ 2

where: *F*_RA_ = recovered fraction in the enhanced recovery stage (%); AC = coded alkali concentration; BSC = coded biosurfactant concentration; BPC = coded biopolymer concentration; and ACBSC = interaction between the coded alkali concentration and the coded biosurfactant concentration.

According to the results presented in Table 6, it can be observed that the proposed model is statistically significant, as the calculated *F*-value (24.138) is greater than the tabulated *F*-value (6.163) in the analysis of variance (ANOVA). Furthermore, the model does not exhibit a lack of fit, since the *p*-value for the lack of fit (LOF) is greater than 0.05.

**Table 6:** Analysis of Variance (ANOVA) for the Influence of the Factors on the Enhanced Oil Recovery Fraction

source of variation	sum of squares	degrees of freedom	mean square	*F*-test	*p*-value
model	780.301	6	130.050	24.138	0.004
residue	21.551	4	5.388		
lack of fit	21.269	2	10.635	6.136	0.130
pure error	0.282	2	0.141		
total	801.852	10			

The regression model obtained shows a good fit and is statistically significant. Figure 2B demonstrates a strong correlation between the experimental results (recovered fraction) and the mathematical model as evaluated by ANOVA.

Analyzing the response surface shown in Figure 3A, it can be stated that, regardless of the alkali concentration (from the minimum to the maximum coded value) within the range of 1200–4500 mg L^–1^, it is the biosurfactant concentrations that will increase or decrease the recovered oil fraction. The red region represents the surface with the highest response for the recovered fraction, and the dark green region represents the lowest response. The recovered fraction varied from 36.18% when using the Bio-ASP1 fluid (low concentrations of biosurfactant and biopolymer) to 63.15% when using the Bio-ASP8 fluid (high concentrations of biosurfactant and biopolymer).

**Figure 3:**
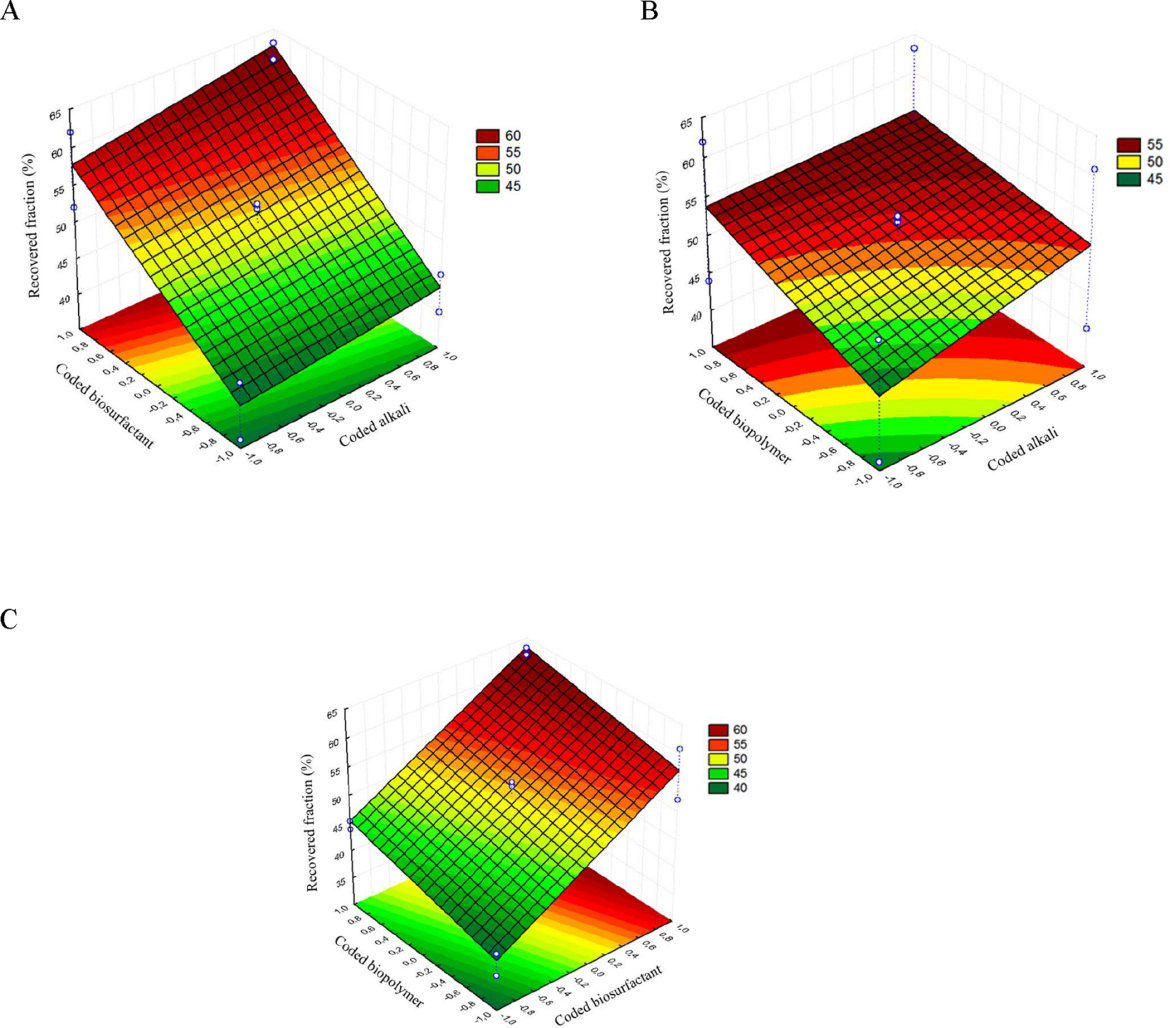
Response surfaces illustrating the relationship and synergistic effects of the interactions between the Bio-ASP solution components on the recovered oil fraction. The surfaces were generated from the mathematical model, with red areas indicating the highest recovery fraction and dark green areas indicating the lowest. (A) Response surface for the alkali and biosurfactant interaction; (B) response surface for the alkali and biopolymer interaction; (C) response surface for the biosurfactant and biopolymer interaction.

The chemical nature of the biosurfactant directly influences its ability to reduce IFT and modify wettability, which are key parameters in capillary-driven displacement processes.^30^

Observing Figure 3B, it can be stated that regardless of the alkali concentration used in the process, from the minimum to the maximum coded value, it is the biopolymer concentrations that cause a variation in the recovered oil fraction.

Laboratory evaluation using core samples and reservoir fluids should be conducted to select a biopolymer suitable for the specific reservoir in question. Additionally, proper selection can provide an indication of mobility control, blockage trends, and adsorption losses. Significant interactions between the porous medium and the molecules of the transported biopolymers can occur, causing the biopolymer to be retained on the porous surface. This retention may reduce the efficiency of biopolymer injection, as well as the permeability of the rock.^30,31^ For biopolymer concentrations ranging from the central point to the maximum coded value, the highest recovery fractions are obtained. In contrast, for biopolymer concentrations from the central point to the minimum coded value, the recovered fraction is low.

The response surface shown in Figure 3C illustrates the relationship between biopolymer and biosurfactant concentrations and the response variable. It can be observed that the biosurfactant concentration is the most statistically relevant independent variable when compared to the biopolymer concentration. For biopolymer concentrations ranging from the minimum to the maximum coded value, lower recovery fractions are obtained when compared to the biosurfactant concentrations.

According to Assis et al.,^21^ the viscosity of the aqueous phase through the addition of the biopolymer effectively contributes to EOR, and the presence of a surfactant associated with it may contribute more effectively to the success of the operation.

Based on the information provided by statistical planning, the Bio-ASP8 fluid presents the ideal formulation for achieving the maximum recovered oil fraction. The synergy presented by the components of the Bio-ASP solution was enhanced by high alkali, biopolymer, and biosurfactant concentrations, which likely prevented or reduced the adsorption of this biomaterial in the simulated reservoir.

The enhanced recovery factor observed with Bio-ASP solutions at higher biosurfactant concentrations can be attributed to surfactin’s strong capacity to substantially lower the oil–water IFT, thereby overcoming the capillary forces responsible for trapping residual oil. Wang et al.^32^ reported that an ASP formulation containing surfactin reduced IFT from approximately 23.5–4.6 mN m^–1^, leading to improved oil recovery when surfactin was employed as a coupling surfactant. Similarly, Fernandes et al.^33^ demonstrated that surfactin produced by *Bacillus subtilis* RI4914 was capable of mobilizing up to 40% of residual oil in porous media, particularly at elevated biosurfactant concentrations.

Xanthan gum primarily enhances the recovery factor through its rheological properties: its non-Newtonian behavior increases the viscosity of the aqueous phase, thereby improving the mobility control and sweep efficiency. Studies combining natural surfactants with xanthan gum and silica nanoparticles have shown that this formulation promotes emulsification, stabilizes displacement fronts, and increases oil recovery in core-flooding experiments.^34^ Moreover, green SP (surfactant–polymer) systems employing xanthan in carbonate cores underscore the significance of surfactant–polymer synergy for tertiary recovery.^35^

The synergistic interaction between surfactin and xanthan gum in Bio-ASP formulations may therefore account for the high recovery observed in the optimized system, where IFT reduction and mobility control act concurrently. By contrast, the contribution of alkali appears to be secondary in this context, likely because surfactin already provides substantial IFT reduction; the alkali may instead serve to stabilize surfactin, maintain a favorable pH, or reduce surfactant adsorption onto rock surfaces. Collectively, these mechanisms indicate that oil recovery in Bio-ASP formulations is governed primarily by interfacial modification and mobility control rather than by the alkaline effect alone.

The results obtained in this study are directly relevant to the practical application of Bio-ASP systems in the petroleum industry. The high oil recovery achieved by formulations containing biosurfactants and biopolymers demonstrates the potential of this approach as an environmentally sustainable alternative to conventional ASP flooding, which relies on synthetic and often environmentally persistent chemicals. In particular, the use of renewable substrates for producing surfactin and xanthan gum offers a cost-effective and scalable route for large-volume field applications. This is especially attractive for mature or low-productivity reservoirs, where incremental recovery is vital for extending the field life and reducing the need for new developments. Moreover, the observed synergistic effects between biosurfactant and biopolymer highlight the opportunity to design customized Bio-ASP formulations tailored to specific reservoir conditions, thereby improving efficiency while aligning with global goals of a sustainable energy transition.

Although the surfactin concentration showed the most statistically significant effect on reducing IFT, the contribution of xanthan gum is equally key to increasing viscosity and controlling mobility. The synergistic interaction between both components ensures an efficient increase in the recovery factor, demonstrating that both interfacial modification and flow control mechanisms are decisive in tertiary oil recovery.

## 4 Conclusions

This study demonstrated the technical viability of a novel Bio-ASP formulation composed of sodium carbonate, surfactant, and xanthan gum for EOR in sandstone reservoirs. The factorial design analysis revealed that surfactin concentration had the strongest effect on oil recovery, increasing the recovered fraction by up to 18.1%, while xanthan gum contributed an additional 6.3% through viscosity enhancement and mobility control. In contrast, the alkali concentration had a smaller but still positive effect of approximately 3.8%.

The optimized Bio-ASP (Bio-ASP8) achieved a maximum tertiary recovery of 63%, reaching 95% of the original oil in place. Although these values are promising, a direct comparison with conventional ASP systems (e.g., HPAM) was not conducted in this study; therefore, conclusions regarding the relative superiority should be interpreted with caution.

The optimized Bio-ASP solution achieved a recovered oil fraction of up to 63% in the enhanced stage, demonstrating its high potential to improve the sweep efficiency, reduce IFT, and enhance displacement mechanisms in porous media. These results are particularly relevant when compared to those of conventional ASP formulations, which often rely on synthetic and environmentally persistent chemicals.

Importantly, the use of a biosurfactant and biopolymer derived from renewable sources makes the proposed Bio-ASP strategy more environmentally friendly and economically attractive. This contributes to the advancement of more sustainable EOR technologies aligned with the principles of energy transition, particularly for mature or low-productivity fields, where incremental recovery is vital.

The results show that EOR in Bio-ASP formulations arises from a synergistic mechanism between the interfacial activity of surfactin and the rheological modification induced by xanthan gum. This interaction improves mobility control and progressively increases the capillary number, reducing residual oil saturation. The injection pressure values confirmed stable slug propagation and an efficient sweep without injectivity losses. The desaturation curve behavior follows classical trends with limited mobilization at low capillary numbers, a pronounced reduction in Sor in the transition region, and responses characteristic of the viscous regime at higher *N*_c_. Overall, the system exhibits consistent capillary desaturation driven by combined interfacial and rheological effects.

Although IFT values did not reach the ultralow range (<10^–3^ mN·m^–1^) typically associated with microemulsion formation, recovery performance remained high. This observation is consistent with recent findings demonstrating that oil mobilization can be dominated by polymer-induced mobility control and viscoelasticity, rather than IFT reduction alone.^34,35^ The xanthan gum used in this study significantly increased the viscosity of the displacement fluid, improving the sweep efficiency by suppressing viscous instability and increasing macroscopic displacement. For future work, it is interesting to perform flooding tests with only the polymer to confirm a synergistic contribution with the other compounds in the Bio-ASP solution.

The novelty of this work lies in the integration of biosurfactant and biopolymer components, both derived from renewable sources, into a ternary ASP system. Unlike conventional ASP flooding, the Bio-ASP approach reduces reliance on synthetic and environmentally persistent chemicals while maintaining a high technical performance. This highlights the dual contribution of the study: advancing EOR efficiency through interfacial and rheological optimization and promoting a more sustainable, scalable, and economically viable strategy for mature or low-productivity oil fields.

## List of Acronyms

AC: Alkali concentration (coded variable)
ANOVA: Analysis of variance
ASP: Alkaline–surfactant–polymer
Bio-ASP: Biobased alkaline–surfactant–polymer
BPC: Biopolymer concentration (coded variable)
BSC: Biosurfactant concentration (coded variable)
DOE: Design of experiments
EOR: Enhanced oil recovery
FRA: Recovered fraction in the enhanced stage
IFT: Interfacial tension
OOIP: Original oil in place
PV: Pore volume
RFA: Recovery factor in the bio-ASP stage
RFW: Recovery factor in the water flooding stage

## References

[1] Quintella C. M., Rodrigues P. D., Ramos-de-Souza E., Carvalho E. B., Nicoleti J. L., Hanna S. A. (2025). Integration of EOR/IOR and Environmental Technologies
in BRICS and nonBRICS: A Patent-Based Critical Review. Energy Reports.

[2] U.S. Energy Information Administration (EIA) . Oil Producers and Consumers. https://www.eia.gov/tools/faqs/faq.php?id=709&t=6 (accessed 10 February 2025).

[3] WATT-Energy Institute. Statistical Review of World Energy 2024. Heriot Watt University, 2024. https://www.energyinst.org/statistical-review (accessed 13 February 2025).

[4] Fincham G. (2024). “A
World Not Our Own to Define”: Ecological Solutions to Global
Catastrophe in the Works of Barry Lopez. English
Academy Review.

[5] Li G., Zhou Z., Fan J., Zhang F., Zhao J., Zhang Z., Ding W., Zhang L., Zhang L. (2024). Study on Microscopic
Oil Displacement Mechanism of Alkaline–Surfactant–Polymer
Ternary Flooding. Materials.

[6] Zhong H., Shi B., Bi Y., Cao X., Zhang H., Yu C., Tang H. (2025). Interaction of Elasticity
and Wettability on Enhanced Oil Recovery
in Viscoelastic Polymer Flooding: A Case Study on Oil Droplet. Geoenergy Science and Engineering.

[7] Rock A., Hincapie R. E., Tahir M., Langanke N., Ganzer L. (2020). On the Role
of Polymer Viscoelasticity in Enhanced Oil Recovery: Extensive Laboratory
Data and Review. Polymers.

[8] Golab E. G., Parvaneh R., Riahi S., Vatankhah-Varnosfaderani M., Nakhaee A. (2024). Study on Interfacial
Tension, Wettability and Viscosity
in Different Salinities of Synthesized a New Polymeric Surfactant
for Improving Oil Recovery. Sci. Rep.

[9] Liu Y., Zhang X., Wu X., Hou Z., Wang M., Yang E. (2024). Research on Microbial Community Structure
in Different Blocks of
Alkaline–Surfactant–Polymer Flooding to Confirm Optimal
Stage of Indigenous Microbial Flooding. Applied
Sciences.

[10] Al-Araimi, S. H. ; Al-Bahry, S. N. ; Al-Wahaibi, Y. M. Using Fungal Biopolymers for Enhanced Oil Recovery. In Fungal Biopolymers and Biocomposites; Springer Nature: Singapore, 2022; pp 85–103. 10.1007/978-981-19-1000-5_6.

[11] Aboelkhair H., Diaz P., Attia A. (2022). Biosurfactant Production
Using Egyptian
Oil Fields Indigenous Bacteria for Microbial Enhanced Oil Recovery. J. Pet. Sci. Eng..

[12] Ashraf
Soliman A., El-hoshoudy A. N., Attia A. M. (2020). Assessment of Xanthan
Gum and Xanthan-g-Silica Derivatives as Chemical Flooding Agents and
Rock Wettability Modifiers. Energies nouvelles.

[13] An E. (2018). Synthesis
and Evaluation of Xanthan-G-Poly (Acrylamide) CoPolymer for Enhanced
Oil Recovery Applications. Pet. Petrochem. Eng.
J..

[14] Sharma J., Kapley A., Sundar D., Srivastava P. (2022). Characterization
of a Potent Biosurfactant Produced from Franconibacter Sp. IITDAS19
and Its Application in Enhanced Oil Recovery. Colloids Surf., B.

[15] Lourdes R. S., Cheng S. Y., Chew K. W., Ma Z., Show P. L. (2022). Prospects
of Microbial Enhanced Oil Recovery: Mechanisms and Environmental Sustainability. Sustainable Energy Technologies and Assessments.

[16] Kaboli A., Jafari A., Azarhava H., Mousavi S. M. (2022). Performance Evaluation
of Produced Biopolymers by Native Strains on Enhanced Oil Recovery. J. Appl. Polym. Sci..

[17] El-hoshoudy A. N. (2022). Experimental
and Theoretical Investigation for Synthetic Polymers, Biopolymers
and Polymeric Nanocomposites Application in Enhanced Oil Recovery
Operations. Arab J. Sci. Eng..

[18] Soares C. C., Adrielly S. A. D. A., Gabriela F. D. F., Almeida A. F. D., Janice I. D., Ana K. D. C. L. L. (2018). Biosurfactant Production by Bacillus Subtilis UFPEDA
86 Using Papaya (Carica Papaya L.) Waste as Substrate: Viability Studies
and pH Influence of the Culture Medium. Afr.
J. Biotechnol..

[19] Charoentanaworakun C., Srisuriyachai F., Assabumrungrat S., Soottitantawat A. (2023). Performance
and Salinity Tolerance of Palm Oil-Derived Anionic Biosurfactant and
Synthetic Surfactant for Waxy Oil Recovery in Sandstone Reservoirs. Energy Fuels.

[20] Câmara J. M. A., Sousa M. A. S. B., Neto E. B., Oliveira M. C. A. (2019). Application
of Rhamnolipid Biosurfactant Produced by Pseudomonas aeruginosa in
Microbial-Enhanced Oil Recovery (MEOR). J. Pet.
Explor. Prod. Technol..

[21] Assis D., Brandão L. V., De Sousa Costa L. A., Figueiredo T. V. B., Sousa L. S., Padilha F. F., Druzian J. I. (2014). A Study
of the Effects
of Aeration and Agitation on the Properties and Production of Xanthan
Gum from Crude Glycerin Derived from Biodiesel Using the Response
Surface Methodology. Appl. Biochem. Biotechnol..

[22] Zhang J., Ge D., Wang X., Wang W., Cui D., Yuan G., Wang K., Zhang W. (2021). Influence of Surfactant and Weak-Alkali
Concentrations on the Stability of O/W Emulsion in an Alkali-Surfactant–Polymer
Compound System. ACS Omega.

[23] Ramos S. E., Rodrigues P. D., Sampaio I. C. F., Bacic E., Crugeira P. J. L., Vasconcelos A. C., Dos Santos Silva M., Dos Santos J. N., Quintella C. M., Pinheiro A. L. B., Almeida P. F. D. (2022). Xanthan
Gum Produced by Xanthomonas Campestris Using Produced Water and Crude
Glycerin as an Environmentally Friendlier Agent to Enhance Oil Recovery. Fuel.

[24] Marques L. S., Rodrigues P. D., Simonelli G., Assis D. D. J., Quintella C. M., De Carvalho Lima Lobato A. K., Maria Cordeiro De Oliveira O., Lobato Dos Santos L. C. (2023). Optimization of Enhanced Oil Recovery Using ASP Solution. Heliyon.

[25] Quintella, C. M. ; Rodrigues, P. D. ; Silva, H. R. ; Carvalho, E. B. ; Souza, E. R. D. ; Santos, E. ; Nicoleti, J. L. ; Hanna, S. A. Smart Water as a Sustainable Enhanced Oil Recovery Fluid: Covariant Saline Optimization. In Offshore Technology Conference Brasil; OTC: Rio de Janeiro, Brazil, 2023. Paper D031S032R003. DOI: 10.4043/32800-MS.

[26] Kumar
Pandey R., Gandomkar A., Vaferi B., Kumar A., Torabi F. (2023). Supervised Deep Learning-Based Paradigm to Screen the
Enhanced Oil Recovery Scenarios. Sci. Rep.

[27] Zivar D., Pourafshary P., Moradpour N. (2021). Capillary Desaturation Curve: Does
Low Salinity Surfactant Flooding Significantly Reduce the Residual
Oil Saturation?. J. Petrol Explor Prod Technol..

[28] Berthet, H. ; Rivenq, R. Capillary Desaturation Curves and Insights on Trapped Oil at the Pore Scale, in Water-Wet and Oil-Wet Sandstones. In 80th EAGE Conference and Exhibition; European Association of Geoscientists and Engineers (EAGE): Copenhagen, Denmark, 2018. DOI: 10.3997/2214-4609.201800842.

[29] Shakeel M., Samanova A., Pourafshary P., Hashmet M. R. (2021). Capillary Desaturation
Tendency of Hybrid Engineered Water-Based Chemical Enhanced Oil Recovery
Methods. Energies.

[30] Ni’matuzahroh, Sari S. K., Trikurniadewi N., Ibrahim S. N. M. M., Khiftiyah A. M., Abidin A. Z., Nurhariyati T., Fatimah (2020). Bioconversion of
Agricultural Waste Hydrolysate from Lignocellulolytic Mold into Biosurfactant
by Achromobacter Sp. BP(1)­5. Biocatalysis and
Agricultural Biotechnology.

[31] She H., Kong D., Li Y., Hu Z., Guo H. (2019). Recent Advance
of Microbial Enhanced Oil Recovery (MEOR) in China. Geofluids.

[32] Wang, D. ; Zhang, Y. ; Yongjian, L. ; Hao, C. ; Guo, M. The Application of Surfactin Biosurfactant as Surfactant Coupler in ASP Flooding in Daqing Oil Field. In SPE Asia Pacific Oil and Gas Conference and Exhibition; Society of Petroleum Engineers (SPE): Jakarta, Indonesia, 2009; Paper 119666-MS, DOI: 10.2118/119666-MS.

[33] Fernandes P. L., Rodrigues E. M., McInerney M. J., Tótola M. R. (2024). Microbial
Enhanced Oil Recovery: Use of Metabolic Products of Bacillus Subtilis
RI4914 in Non-Consolidated Porous Media and Influence of Environmental
Parameters. Colloids Surf., A.

[34] Saha R., Uppaluri R. V. S., Tiwari P. (2019). Impact of
Natural Surfactant (Reetha),
Polymer (Xanthan Gum), and Silica Nanoparticles To Enhance Heavy Crude
Oil Recovery. Energy Fuels.

[35] Haq B. (2021). Green Enhanced
Oil Recovery for Carbonate Reservoirs. Polymers.

